# User experience and real-world implementation of a peer-integrated digital health intervention for substance use disorder

**DOI:** 10.21203/rs.3.rs-9497767/v1

**Published:** 2026-05-12

**Authors:** Talia Feldman, Reynalde Eugene, Nirzari Kapadia, Premananda Indic, Jazmin Hampton, Stephanie Carreiro

**Affiliations:** University of Massachusetts Chan Medical School; University of Massachusetts Chan Medical School; University of Massachusetts Chan Medical School; The University of Texas at Tyler; University of Massachusetts Chan Medical School; University of Massachusetts Chan Medical School

## Abstract

Digital health interventions (DHIs) are a promising tool for supporting recovery from substance use disorder (SUD), yet implementation in vulnerable populations remains a concern. We conducted a 30-day observational study of Realize, Analyze, Engage (RAE) Health, a wearable sensor based DHI in dyads of clients with SUD (N = 75) and peer recovery professionals (N = 16) from 11 US outpatient recovery programs. Outcomes include acceptability, engagement, usability, and sustainability with validated instruments. Clients demonstrated high acceptability (Treatment Acceptability and Preferences Measure score = 3.37/4) and usability (System Usability Scale = 70.9): peers demonstrated high usability (78.2) and favorable acceptability and sustainability. Mean client app connectivity was 13.8/30 days. No significant differences in outcomes were observed by client income, employment, or education. These findings support real-world implementation of a peer-integrated DHI for SUD and suggest that embedding peer support within a DHI may extend reach across socioeconomic backgrounds.

## Introduction

Substance use disorder (SUD) is a significant public health issue, affecting 16.8% of Americans age 12 or older in 2024^1^. It is characterized by continued substance use despite significant clinical and functional impairment as a result of changes in neural circuits^2^. In recent years treatment of SUD has evolved from primarily abstinence-based to holistic and individualized, incorporating FDA-approved medications, evidence-based behavioral therapies, and treatment programs^3^. Along with this evolution has come the advent of novel technology-based treatment interventions, including digital health technologies, which integrate wearable devices and smartphones into treatment strategies^3^. Such digital health interventions (DHIs) have increasingly been recognized and utilized in the assessment and treatment of SUD, expanding access to evidence-based clinical care outside of traditional treatment facilities^4^. They can be harnessed for both the detection and assessment of substance use and their associated behaviors, capturing patient reported data, which enable users to quickly report data on substance use and craving, all while measuring substance use-related physiological data such as heart rate, electrodermal activity, and skin temperature^5^.

While DHIs hold promise to support and accelerate the treatment of SUD, there remain several questions and concerns related to their implementation and equity amongst end-users. While digital systems can collect extensive data on users’ treatment progress, it is unclear which providers are best positioned to review and respond to these data with users. Clinicians are often overwhelmed with data atop their clinical duties, and digital health systems have been associated with increased mental workload and burnout in healthcare professionals^6,7^. Other concerns have been raised over the potential for DHIs to both generate and exacerbate existing health disparities as a result of differential access (i.e., broadband and device access) and use (i.e., digital literacy) across demographic and socioeconomic groups^8,9^. Low confidence in using digital health has been noted as a key barrier in accessing DHIs^10^, with one systematic review finding that effective use of DHIs was less likely for individuals with low incomes, lower levels of education, and those belonging to a minority ethnic group^11^. Given the association of social adversities (including low education, unemployment, and homelessness) and social vulnerability with the presence of SUD^12,13^, as well as the association between low socioeconomic status (SES) and drug-related relapses, overdose, and mortality^14,15^, concerns remain regarding the potential for social determinants of health (SDoH) and SES-related factors to hinder the utilization and effectiveness of DHIs.

Integrating peer recovery professionals into the workflow of a DHI may represent a promising and practical strategy to overcome some of these barriers. In our prior work, we describe the validation of a DHI (Realize, Analyze, Engage or RAE Health) that detects stress and drug craving and deploys real-time support using a wearable sensor and mobile application^16,17^. Data on self-reported stress and craving events were collected alongside continuous physiologic data (e.g., heart rate, accelerometry), and algorithms were built and validated to identify physiologic correlates of stress and craving with classification accuracy of 71.6% and 71.0% respectively. In the original study, individuals with SUD were asked to use the app alone; however, participants who self-initiated a "buddy system" (e.g. partner, child, clinician, or peer who also helped them use the DHI) showed higher engagement and satisfaction with the DHI^18^. Furthermore, we found that users from sites serving a low SES population had lower engagement^18,19^.

In this study, we piloted the RAE Health DHI with a companion app (Connected Health, or cHealth) in a diverse cohort of individuals in recovery for SUD receiving peer recovery services. Building on our prior findings, the cHealth DHI intentionally incorporates a “buddy system” into the new DHI platform, by engaging clients with SUD as dyads alongside their peer recovery professionals, each of whom are connected to the system with a peer companion version of the app. Our objectives were to measure acceptability, usability and engagement with the cHealth system in clients and acceptability, usability, and sustainability with the system in peer recovery professionals. Additionally, we aimed to compare client outcomes based on social determinants of health, including income, employment status, or level of education, as well as characteristics of the peer-client dyad.

## Methods

Study methods are reported in alignment with the Strengthening the Reporting of Observational Studies in Epidemiology (STROBE) guidelines for observational studies^20^.

### Study design

This is a primary analysis of data collected from an observational pilot study that deployed the RAE cHealth DHI in dyads of clients with SUD and their respective peer recovery professionals. Dyads were recruited and provided informed consent independently. Dyads were then trained on the use of the RAE cHealth digital intervention system and asked to use the system together for 30 days. Baseline data were collected upon enrollment, and outcome measures to assess acceptability, sustainability, and usability were collected at 30 days. Data regarding app use to assess engagement were collected through the 30-day study period. This study was approved by the UMass Chan Medical School Institutional Review Board, docket # STUDY00000957.

### RAE System

In this study, two separate apps were used: the client facing RAE Health app, and the peer facing companion app, RAE cHealth.

**Figure 1:**
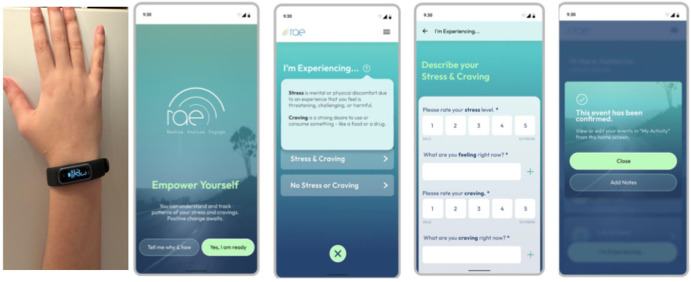
Garmin Vivosmart 4 and screen shots of the client facing RAE health app.

Details of the client facing RAE system have been described elsewhere^17,19^. Briefly, the system is composed of a wearable sensor (Garmin Vivosmart 4)^21^ and a mobile application that runs on a smartphone (iOS or Android, Fig. 1). System end-users (clients) wear the smartwatch-like device, which continuously collects physiologic data including accelerometry, heart rate, and heart rate variability. The system uses a validated machine learning algorithm described previously to detect stress or substance-craving events^16,19,22^. If an event is detected, the user receives a notification on their mobile device, which directs them to the RAE application. There, the user is asked to confirm or deny the event, given the option to provide additional contextual information regarding the event and provided with the opportunity to engage in de-escalation tools such as responding to journaling prompts or completing a mindful breathing exercise.

**Figure 2:**
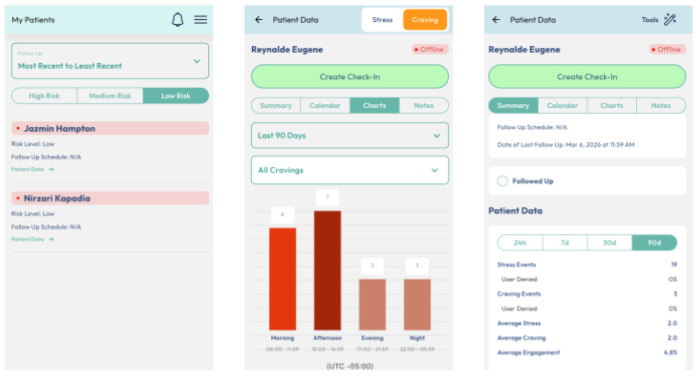
Screenshots of peer facing RAE cHealth app showing client roaster and individual graphic/numeric data displays

The peer-facing part of this system, RAE cHealth app (Fig. 2), was specifically designed for peer recovery professionals to monitor their clients’ stress and craving. The peers were asked to use a companion app, RAE cHealth, and monitor their clients' stress and craving data for 30 days. The RAE cHealth app consists of a dashboard displaying the peer’s client roster, and the peer can designate clients to a certain risk level (i.e. high, medium or low risk). Peers had access to the stress, craving, and connectivity data for each client. Peer providers were given the opportunity in the app to assess clients SDoH needs, document check ins, and add notes regarding each client session and document their progress. During the study period, peers were asked to monitor clients' stress and craving data in the app once per day. All other features were available for them to use at their discretion.

### Setting

Participants were recruited to pilot the cHealth system from 11 outpatient, peer-recovery based treatment programs from across the United States. These included privately-owned medical clinics offering substance use-related counseling services, community-based SUD treatment clinics and recovery centers, mental health treatment facilities, and virtual behavioral support and recovery services. Study procedures were offered in-person to sites local to the study team, and virtually to all other participants. Study procedures and data collection were conducted remotely via phone call and/or videoconference (Zoom). Self-reported survey data, including baseline demographic and SDoH and follow-up outcomes from all participants (clients and peers), were collected and managed using electronic data capture tools on REDCap, a secure, web-based software platform^23,24^. Clients' sensor-based data including physiologic data and user-reported event annotations (i.e., stress and craving) were collected remotely through the HIPAA-compliant, cloud-based RAE system during the active monitoring period.

### Participants and Recruitment

Clients with SUD were recruited as part of dyads with their established peer recovery professionals. To be eligible, clients must have been diagnosed with SUD, been 18 years of age or older, and engaged in treatment for SUD using peer recovery services. Additionally, clients must have had access to a smartphone with iOS or Android capabilities, been able to read and speak in English, and must not have had limitations of motion on the non-dominant arm on which the wearable sensor would be worn (e.g fracture, amputation). Clients were excluded if unable to consent or if classified as a prisoner. Peer recovery professionals were enrolled as the providers in the dyad. To be eligible, peers must have been providing peer recovery services to clients in treatment for SUD, be 18 years of age or greater, able to read and speak in English, and have access to a smartphone with iOS or Android capabilities. Peers were allowed to participate in the study multiple times (with different clients): however, they were only asked to complete the surveys described below on their first round of participation.

Participants were recruited through two methods: site-specific leadership informed potential clients of the study and provided them with an IRB-approved study flyer, and word of mouth referrals were provided from client to client as well as peer to client.

### Variables, data sources, and measurement

Outcomes included acceptability, engagement, sustainability, and usability of the cHealth system. Of note, outcomes were measured across both clients and peer recovery professionals; however, each group had a fundamentally different relationship with the system — clients used the app to self-monitor physiologic and behavioral data as part of their treatment, while peers used it as a clinical tool to monitor client progress and guide their delivery of peer recovery services. To capture these distinct perspectives and roles, outcomes and validated instruments were selected that were most appropriate for each group's context, which in several cases differed between groups.

#### Acceptability

Acceptability is a component of user experience that relates to users’ perceived usefulness and satisfaction with a tool^25^. Acceptability for clients was measured at 30 days using the Treatment Acceptability and Preferences Measure (TAP). TAP is a validated measure that assesses user acceptability and preference for the technology compared to alternative treatment options. Respondents rate the treatment option based on four attributes: acceptability, suitability, effectiveness, and willingness to comply, using a scale from 0 (not at all) to 4 (very much). The mean of the attribute scores is calculated, with higher scores reflecting greater user acceptability^26^. Acceptability for peer recovery professionals was measured using a questionnaire based on the Technology Acceptance Model (TAM) rather than the TAP, as the TAM was developed specifically to assess technology adoption behavior in a provider context^27,28^, which more accurately reflects the peer role within the cHealth system. The TAM questionnaire is a 33-item questionnaire assessing eight dimensions (perceived usefulness, perceived ease of use, attitude, compatibility, subjective norm, facilitators, habits and intention to use) to predict technology use. Each dimension is scored on a scale of 1–7, with higher scores being more favorable^29^.

#### Engagement

Engagement was measured in clients only, as objective usage data were derived from the client-facing wearable sensor and mobile application; no equivalent sensor-based data were available for peer recovery professionals. Engagement for clients was defined as usage of the RAE system, operationalized as sensor connectivity with the mobile app. Throughout the 30-day monitoring period, the hours per day that the RAE app and the sensor were connected and the number of days where there was sensor data detected were measured. For engagement outcomes, data were analyzed both continuously (i.e., mean number of days and hours per day the RAE application was connected throughout the 30-day active protocol) and dichotomously (i.e., high vs. low level of application use) to test for association with social determinants of health and client-peer differences in demographics. A high level of application use was defined as use for greater or equal to 15 days of use during the 30-day protocol ( ≧ 50%), while a low level was defined as anything less, consistent with prior literature^19^. Moreover, the final day within the 30-day period on which more than one hour of sensor data was recorded was identified for each participant. This measure was used to construct Kaplan-Meier curves to visualize the probability of sustained app connectivity across different SDoH subgroups.

#### Sustainability

Sustainability is defined as “the extent to which a newly implemented treatment or intervention is maintained within a health system's existing operations”^30^. Sustainability was measured at 30-days in the peer recovery professional participant group through the use of the Normalization Measure Development Questionnaire (NoMAD)^31^. The NoMAD is a validated 23 item questionnaire that measures perception on the implementation and normalization of a new practice or technology. It contains 20 items assessing the full range of normalization constructs and 3 items on participants' perception on implementation. Implementation is measured using the following 4 domains; coherence, cognitive participation, collective action, and reflective monitoring. Each item is scored using a 5-point Likert scale.

#### Usability

Usability is one component of the user experience, defined as “how easily a user can accomplish their goals when using a service”^32^. Usability was measured from both clients and peers at 30 days using the System Usability Scale (SUS), a reliable and validated tool frequently utilized to assess digital tools^33^. The SUS is a 10-item questionnaire composed of 5 positive and 5 negative statements to which respondents rate the degree to which they agree with each statement on a Likert scale. Total scores range from 0-100, with mean scores greater or equal to 69 considered to indicate a usable tool^34,35^. For usability, SUS was analyzed both continuously (0-100 scale) and dichotomously using the score of ≥ 69 cutoff (high vs. low usability).

#### Social Determinants of Health

All client outcome measures were compared based on SES-related SDoH, which was approximated through the variables education, income, and employment status as obtained at baseline. Education was collected by level, from 8th grade or less to graduate or professional degree. Income was collected as household income within one of five brackets ranging from $0 to $92,000+, and coded as above or below the federal poverty level^36^. Employment status was defined within the past 3 years and collected as ten categories ranging from full-time employment to homemaker. For the statistical analysis, income was analyzed dichotomously as above or below the federal poverty line. Education level was categorized into three groups for analysis: high school diploma or less, some college or a non-four-year degree (e.g., associate’s, technical/trade school), and a Bachelor’s degree or higher (e.g., graduate, master’s, doctorate). Employment status was categorized into three groups for analysis: full-time (35 + hours), unemployed, and other (e.g., part-time, student, retired). These groupings allowed for a larger sample size within each category, increasing statistical power to enable the detection of significant differences.

Other SDoH used to characterize the population included food insecurity and disability status. Food insecurity was assessed using three items adapted from the USDA Household Food Security Survey Module^37^, capturing food supply adequacy, meal affordability, and meal skipping due to financial constraints; participants endorsing one or more items were classified as food insecure. Disability status was assessed using five items drawn from the CDC Disability and Health Data System^38^, querying difficulty with walking, lifting, seeing, hearing and cognition on a four-point scale from no difficulty to unable to do; participants reporting difficulty in at least one domain were classified as having a disability.

### Bias

In order to reduce selection bias, study site leadership were asked to advertise study to all eligible clients, not just individuals they thought would be good candidates. Clients were informed that their decision regarding participation (and use of the app) would not have a negative impact on the care they otherwise received.

### Study size

This was a feasibility and user-centered outcomes study, so the sample size was selected to ensure an adequate number of participants would complete the full protocol to support stable estimates of engagement, acceptability, and implementation outcomes. We initially planned to enroll 60 participants to yield at least 40 protocol completers, conservatively assuming up to 40% attrition, which is commonly observed in digital health studies in substance use populations^19,39,40^. To further protect against loss to follow-up and incomplete data, and to allow participation of clients who were already in the enrollment pipeline, we ultimately enrolled 75 participants.

#### Statistical analysis

Descriptive statistics were calculated for outcome measures, including acceptability, engagement, usability, and sociodemographic characteristics. These outcomes were compared across subgroups of interest: social determinants such as level of education, income, and employment status as well as client-peer demographic differences. Hypothesis testing was conducted to assess differences in each outcome by subgroup.

For continuous variables (e.g., engagement measured by number of days the application was used, System Usability Scale [SUS] scores, and Treatment Acceptability and Preferences [TAP] scores), Student's t-test (for two groups) and analysis of variance (ANOVA) (for more than two groups) were used when data were normally distributed. When normality assumptions were not met, the Wilcoxon rank-sum test (two groups), Kruskal-Wallis H test (greater than two groups) and Spearman correlation test (two continuous variables) were used. Normality of continuous variables was assessed using the Shapiro-Wilk test.

For categorical variables (e.g., education, income, employment status, engagement categorized as high vs. low use, and SUS scores categorized as high vs. low), Pearson’s chi-squared test or Fisher’s exact test was used, depending on expected cell counts. All statistical analyses were conducted using R Statistical Software (*R Core Team*, 2021, Version 4.5.1). Missing data were handled using listwise deletion, such that observations with missing values were excluded from the specific analysis in which they occurred.

## Results

### Sample characteristics

**Figure 3:**
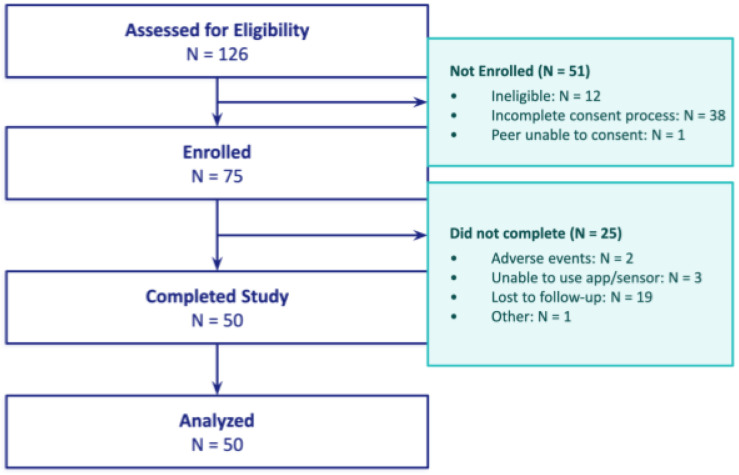
Study pariticipant Flowchart

A total of 126 potential clients were screened, 75 clients were consented between April 2023 to January 2026, and 50 participants (67%) completed the 30-day protocol (Fig. 3). Demographic characteristics of the study sample are presented in Table 1. The client sample had a mean age of 44.5 years (SD = 11.1), 55% were female, 68% self-identified as Black or African American, and 56% resided in the Northeast. The majority (65%) had a mobile device running an Android operating system, with the rest using iOS. In the client population, 63% had household incomes below the poverty line, 44% had full-time employment with 20% of subjects being unemployed, and 34.7% had formal education beyond the high school level. The sample also had 61% clients experiencing food insecurity and 48% identifying as disabled. The most common substance for which participants were in treatment was opioids (42.7%), followed by alcohol (26.7%) and tobacco (13.3%). Most participants had been in treatment for SUD for less than 1 year (81.3%). Client characteristics compared by their protocol completion status are presented in the Supplemental Table. There were no significant demographic differences between those who completed the protocol and those who did not.

A total of 16 peers enrolled (some peers enrolled with multiple clients; hence the lower number of peers compared to clients). The peer sample had a mean age of 47.6 years (SD = 11.5), 69% were female, 63% identified as White and 56% resided in the Northeast. The majority (93%) had a mobile device running an iOS operating system and the mean time working as a peer was 3.4 years. The median number of clients per peer was 1, with 81% (N = 13) peers having less than 5 clients while 13% (N = 3) peers having 5 or more clients in the study.

### Acceptability (Clients and Peers)

**Figure 4:**
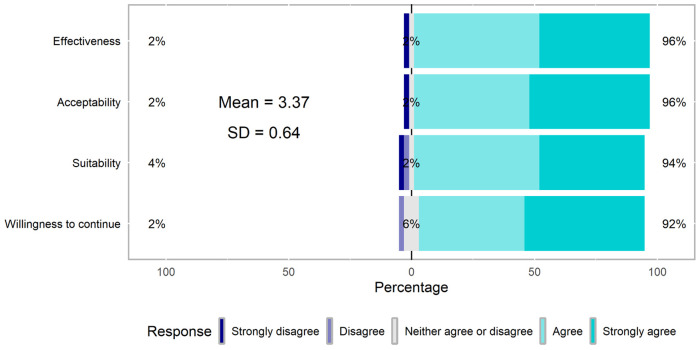
Treatment Acceptability and Preferences Measure (TAP) for Clients

The RAE tool demonstrated high acceptability among clients, with a mean TAP score of 3.37/4 (SD = 0.64). The median score was 3.25, with scores ranging from 0.25 to 4.00 (Fig. 4). TAP scores of clients did not differ significantly by education level (Kruskal–Wallis χ^2^ = 0.04, df = 2, p = 0.98), income status relative to the poverty line (Wilcoxon rank-sum W = 163.5, p = 0.54), or employment category (Kruskal–Wallis χ^2^ = 0.54, df = 2, p = 0.76).

**Figure 5:**
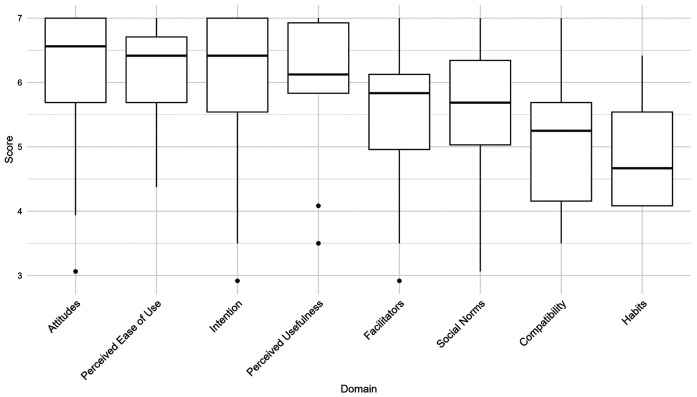
Distribution of Technology Acceptance Model (TAM) Questionnaire for Peers Across Eight Domains

Scores on the Technology Acceptance Measure collected from peers indicated generally favorable perceptions of the technology (Fig. 5). The dimensions of attitudes, perceived ease of use, intention to use and perceived usefulness demonstrated the highest median scores. In contrast, compatibility and habit domains showed lower median scores and greatest variability. TAP scores of clients were not significantly associated with client-peer differences with respect to age (Spearman correlation r = 0.19, p = 0.19), sex (Wilcoxon rank-sum W = 244.5, p = 0.51) or race (Wilcoxon rank-sum W = 57, p = 0.27).

### Engagement (Clients)

Among clients who completed the 30-day protocol, mean app use was 6.6 hours per day and mean sensor connectivity was 13.8 days out of the 30-day study period. Engagement patterns revealed two distinct usage groups: twenty-one participants (42%) demonstrated high engagement, defined as sensor connectivity on 15 or more days, with an average daily connectivity of 8 hours, while thirty-eight participants (54%) exhibited lower engagement, connecting the sensor for fewer than 15 days out of 30, with substantially lower average daily connectivity (2.7 hours per day). Findings were similar when all enrolled clients who used the app were included in the analysis (N = 59, mean connectivity = 11.8 days). No significant associations were observed between study engagement and social determinants of health such as education (χ^2^(2) = 3.13, p = 0.21), income (χ^2^(1) = 0.38, p = 0.54) or employment (χ^2^(2) = 0.41, p = 0.81). Similarly, no significant associations were observed between study engagement and client-peer differences with respect to age (Wilcoxon rank-sum W = 297.5, p = 0.18), sex (χ^2^(1) = 2.42, p = 0.12) and race (χ^2^(1) = 0.21, p = 0.65). Kaplan-Meier curves illustrating the probability of sustained app connectivity across SDoH subgroups are presented in Supplemental Figs. 1, 2 and 3. Overall, these demonstrate a decline in connectivity over the 30-day period, indicating that participants were progressively less likely to remain consistently connected to the app over time. Differences in the curves between subgroups suggest that certain SDoH characteristics such as low income, unemployment and lower education may be associated with earlier disengagement. However, no statistically significant difference was observed for the same.

### Sustainability (Peers)

**Figure 6:**
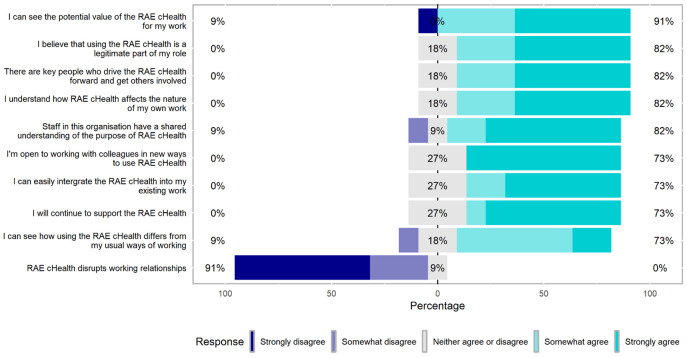
Normalization Measure Development Questionnaire (NoMAD) Responses from Peers- Part 1

**Figure 7:**
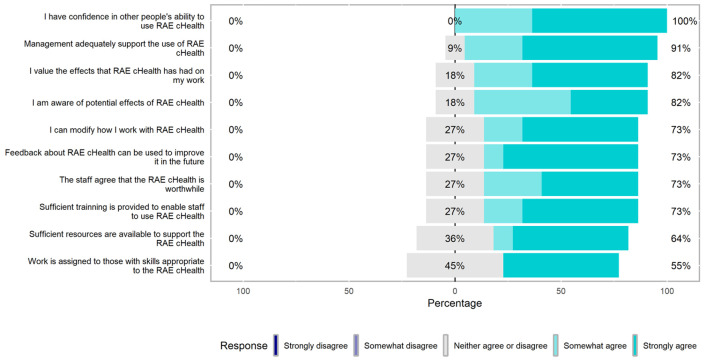
Normalization Measure Development Questionnaire (NoMAD) Responses from Peers- Part 2

The Normalization Measure Development Questionnaire (NoMAD) data collected from the peers showed overall favorable perceptions of the RAE cHealth system (Figs. 6 and 7). Most peers (55–100%) somewhat or strongly agreed to positive statements - for example, 91% somewhat or strongly agreed they could see its potential value and they could easily integrate it into their existing work. Moreover, 82% peers somewhat or strongly agreed that the RAE system is worthwhile, they valued its effects on their work and that feedback could improve it in the future, while fewer (64%) peers somewhat or strongly agreed that sufficient resources were available and that work is assigned to those with appropriate skills. Notably, 91% strongly or somewhat disagreed that RAE cHealth disrupts working relationships.

### Usability (Clients and Peers)

**Figure 8:**
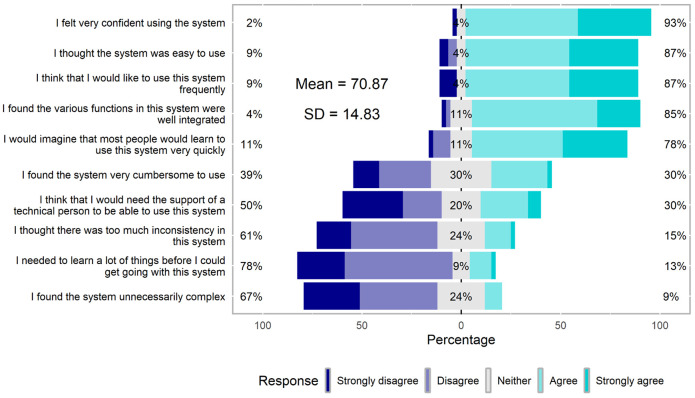
System Usability Scale (SUS) Responses - Clients

The mean SUS score for clients was 70.9/100 (SD = 14.8), with a median score of 71.3 (range: 27.5–97.5), indicating that, on average, participants perceived RAE as highly usable (Fig. 8). When categorized by previously established cutoffs, 26 participants (56.5%) rated the tool as having high usability (SUS ≥ 69), whereas 20 participants (43.5%) reported lower usability (SUS < 69). SUS scores did not differ significantly by education level (*F*(2, 43) = 0.88, *p* = 0.42), income status relative to the poverty line (Welch’s *t*(17) = 0.58, *p* = 0.57), or employment category (*F*(2, 43) = 0.85, *p* = 0.43).

**Figure 9:**
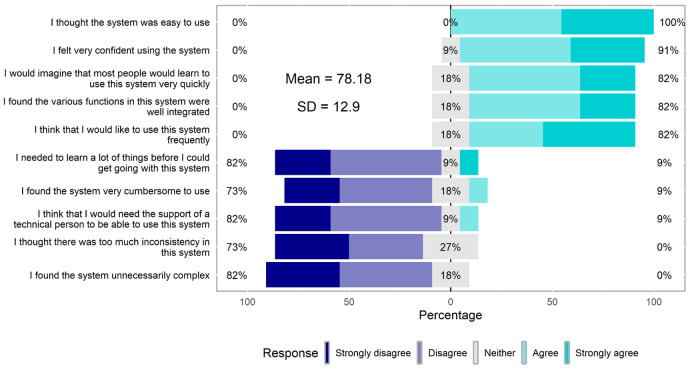
System Usability Scale (SUS) Responses - Peers

Peers reported higher perceived usability of the system than clients with a mean SUS score of 78.2/100 (SD = 12.9), with a median score of 72.5 (range: 62.5–95.0) (Fig. 9). Moreover, 72.7% (N = 8) of peers fell into the high usability group (SUS ≥ 69), while 27.3% (n = 3) were categorized as low usability (SUS < 69). No significant associations were observed between peer SUS score categories and client-peer differences with respect to age (Wilcoxon rank-sum W = 303.5, p = 0.20), sex (χ^2^(1) = 1.56, p = 0.21) and race (χ^2^(1) = 0.67, p = 0.41).

## Discussion

In this observational study we enrolled a racially and socioeconomically diverse sample of N = 75 clients and their peer recovery professionals (N = 16) to assess the usability, acceptability, client engagement and sustainability of the RAE cHealth system. Acceptability in clients was high across all measured domains (effectiveness, acceptability, suitability, and willingness to continue), while peers scored higher in some domains (attitude toward system, intention to use and perceived ease of use) and lower on others (compatibility with current work and fit with current habits). Client engagement was modest despite high acceptability ratings, with completers connecting for a mean of 13.8 out of 30 days contrasting positive user perception with inconsistent behavior. Peers’ responses in the domain of sustainability were encouraging, noting that cHealth did not disrupt working relationships, that cHealth was a reasonable part of their role, and they saw value in cHealth. Usability was high on average in clients and even higher in peers. Importantly, we found no statistically significant difference in acceptability, engagement, or usability based on client SDoH (income, education and employment) supporting the feasibility of this DHI in a broader population.

Our findings demonstrate high acceptability and usability among both clients and peers consistent with prior studies reporting positive user experience with DHIs for SUD, including a review of 32 mHealth intervention for SUD^41^, a feasibility study of an alcohol use disorder app (Step Away) ^42^, and a systematic review by Nesvag, et al.^43^. Despite these favorable perceptions, client engagement was lower than expected- a pattern well documented in the mHealth literature. Pratap et al. reported similar findings in a cross-study evaluation of health-related apps^44^. Wilde et al. found that only 44.0% of participants who downloaded an mHealth intervention for SUD engaged with it meaningfully^45^. This discrepancy may be explained by the distinction between experiential and behavioral components of engagement: while usability and acceptability reflect users’ perception of a technology, sustained engagement requires repeated active behavioral choices that may be difficult to maintain in the context of SUD where competing psychosocial demands are common^46,47^. Furthermore, a decline in user engagement during the protocol is extremely common in studies employing digital health technologies and does not necessarily reflect user experience^48-50^. This gap between perceived value and sustained behavioral use suggests that high acceptability while encouraging may not be sufficient to drive consistent engagement; structured implementation support may be needed to bridge the gap. Peer sustainability data were similarly promising, with NoMAD findings indicating that peers found the system coherent, accepted it as part of their role, and saw potential for long term use. However lower scores in compatibility and resource adequacy domains suggest that role clarity and dedicated implementation resources will be important conditions for normalization. This finding was echoed in a study of telemonitoring scale up within Dutch University Medical Centers in which the provider outlook improved alongside highly structured resources and training^51^.

Notable differences emerged between peer and client participants. Peers consistently rated the system higher among usability and technology acceptance domains, which may in part reflect demographic differences in the groups. Relative to clients, peers had higher levels of education, greater professional stability and were predominantly iOS users, all factors associated with greater digital literacy and confidence. The two groups also differed in their relationship to recovery, with most peers having worked in the space for more than three years, and most clients having been in treatment for less than a year. This disparity might have influenced engagement patterns, as clients in early recovery face greater competing psychosocial demands. Despite these differences, peers viewed the RAE cHealth tool favorably, consistent with Wilde et al.findings that participants viewed a DHI to support (but importantly not replace) interactions with peer recovery professionals^45^.

Despite the socioeconomic vulnerability of our study population with SUD, no significant differences in usability and acceptability were observed by client income, education or employment status. This is notable as it runs counter to prior literature from our group and others suggesting that lower SES is associated with reduced engagement with DHIs for SUD. In our prior work, app users from treatment programs serving lower SES groups demonstrated lower engagement with the standalone RAE Health app^19^, and broader literature has shown lower income, lower education, and minority group membership associated with reduced use (and effectiveness) of DHIs. One potential explanation is the intentional integration of a peer recovery professional into the RAE cHealth experience which might have attenuated some of the SDoH related barriers previously observed. Peer recovery providers who have lived experience with SUD may provide credibility, technical support and motivation that clients with SES challenges may lack when trying to use a tool independently. While this hypothesis requires further exploration, our findings suggest that peer-facilitated DHI may represent a promising strategy for extending the reach of DHIs without widening the digital divide^9^.

Our findings also call into question the concept of intervention dose – that is, what is the level of engagement with a digital tool sufficient to produce positive user experiences and ultimately clinical benefit. Usability and acceptability outcomes were high in our study, even in those who engaged less with the system, raising a question of whether a minimum effective dose exists that maximizes both user engagement and experience. Importantly, user experience and clinical outcomes are distinct entities, and previous literature has shown improved clinical outcomes with higher levels of engagement with digital interventions^52^. Furthermore, defining and measuring such a dose poses its own challenges, as engagement can be characterized by duration (the total length of system use), frequency (how often the system is interacted with), or amount (length of each interaction with the system)^53^. Which of these dimensions is more predictive of outcomes in DHIs remains an open question and should be a target for future study.

### Strengths & Limitations

Our study has many strengths. To our knowledge, this is among the first studies to evaluate user experience outcomes of a peer-integrated wearable DHI for SUD in a diverse, socioeconomically vulnerable population across multiple real-world treatment settings. The use of validated, multidimensional user experience outcome tools (SUS, TAP, NoMAD, TAM) enhances internal validity and comparability with other research. The enrollment of dyads in addition to the use of a commercially available off-the-shelf sensor, reflects real-world implementation conditions and strengthens the ecological validity of our design.

Our study similarly has several limitations. Despite our best efforts, there may be selection bias (i.e, people who consent to be in digital health studies are already more likely to use a digital health intervention, individuals with the most extreme digital inequities may be excluded). The peer group sample size was comparatively smaller, and engagement data were not available for peers. Client engagement was measured by connectivity between sensor, app and cloud: disruptions in connectivity likely underestimated actual/intended use time. Our study did not assess reasons for disengagement (e.g., structural and technical barriers, behavioral and psychosocial factors). Lastly, our study limited outcomes to those related to user experience, therefore, the impact of the cHealth system on outcomes across SES subgroups remains an open questions to be explored in future work.

### Implications and Future Work

Our findings have meaningful implications for the design and implementation of DHIs for SUD. We demonstrate that intentionally embedding peer recovery professionals as active participants in a DHI rather than relying on clients to use the tool on their own is feasible, acceptable, and well- received by both groups. The model harnesses existing peer recovery infrastructure within a DHI, extended the reach of a trusted support system into the moments between in person encounters when triggers are most likely to occur. The finding that user experience outcomes were not significantly different across socioeconomic factors supports the notion that peer-integrated DHIs can broaden access without requiring substantial prior resources. Taken together, these findings support the broader principle that DHIs for SUD are more likely to succeed when they amplify rather than replace human support systems. Future research should explore factors related to the engagement – usability/acceptability discrepancy and assess clinical outcomes amongst vulnerable populations with SUD to ensure that digital interventions for SUD not only promote positive user experience across SES backgrounds, but result in sustained engagement with clinically meaningful results.

## Conclusions

In this pilot study, a peer integrated DHI using a wearable sensor and app demonstrated high usability, acceptability and sustainability across a demographically heterogeneous and socioeconomically vulnerable sample. User experience did not differ by client income, education, or employment status, suggesting that peer-integration may attenuate the SDoH barriers that historically have limited uptake in vulnerable populations. These findings support the larger idea that DHIs are more likely to succeed when used to amplify human support systems as opposed to replacing them. Future research should examine the impact of peer-integrated DHIs on clinical outcomes and the ideal dose of such DHIs.

## Supplementary Material

This is a list of supplementary files associated with this preprint. Click to download.

• cHealthImplementationnpjDMSupplementalMaterials.docx

## Figures and Tables

**Table 1:** Sample demographics

Client demographics (N = 75)		Peer demographics (N = 16)	
	N (%)		N (%)
Age (years)*			
	44.5 (11.1)		47.6 (11.5)
Sex			
Male	33 (45%)	Male	5 (31%)
Female	41 (55%)	Female	11 (69%)
Latino/a			
Yes	6 (8.2%)	Yes	1 (6.3%)
No	66 (90%)	No	15 (94%)
I don’t know	1 (1.4%)	I don’t know	0 (0%)
Race			
Black or African American	50 (68%)	Black or African American	6 (38%)
White	20 (27%)	White	10 (63%)
Other	3 (4.1%)	Other	0 (0%)
US region participant is from			
Northeast	41 (56%)	Northeast	9 (56%)
South	29 (40%)	South	6 (38%)
West	3 (4.1%)	West	1 (6.3%)
Phone operating system			
iOS	25 (35%)	iOS	14 (93%)
Android	46 (65%)	Android	1 (6.7%)
Education level			
Bachelor’s or above	9 (12%)		
Some college	17 (22.7%)		
High school or below	48 (64%)		
Not reported	1 (1.3%)		
Employment in last 3 years			
Full-time	33 (44%)		
Other	26 (34.7%)		
Unemployed	15 (20%)		
Not reported	1 (1.3%)		
Income below poverty line		Clients per peer	
Yes	47 (62.7%)	< 5 clients	13 (81.25%)
No	16 (21.3%)	≥ 5 clients	3 (18.75%)
Not reported	12 (16%)		
Time in treatment		Time working as a peer (years)*	
Less than a year	61 (81.3%)		3.4 (3.12)
More than a year	11 (14.7%)		
Not reported	3 (4%)		

* Mean (SD)

## Data Availability

The data supporting the findings of this study are not publicly available due to proprietary restrictions associated with the Small Business Innovation Research (SBIR) funding mechanism. Data may be available to qualified researchers from the corresponding author upon reasonable request and with appropriate data use agreements and approval from the funding entity. The statistical code for this analysis was implemented in R (Version 4.5.1) using standard packages. Code is available from the corresponding author to qualified academic researchers upon reasonable request.
